# Outcome of the cementless Taperloc stem: a comprehensive literature review including arthroplasty register data

**DOI:** 10.3109/17453674.2011.570668

**Published:** 2011-04-05

**Authors:** Gerold Labek, Stefan Frischhut, Rainer Schlichtherle, Alexandra Williams, Martin Thaler

**Affiliations:** Department of Orthopedic Surgery, Medical University of Innsbruck, Innsbruck, Austria

## Abstract

**Background and purpose:**

The validity of various data sources for the assessment of the outcome quality of medical devices was investigated by comparative analysis of the published data sources available for a sample of implants. It was the aim of the study to determine the performance of this implant and to identify potential bias factors inherent to the various datasets.

**Methods:**

A comprehensive literature search was carried out from English-language, peer-reviewed journals and worldwide reports from national arthroplasty registers. Publications from Medline-listed journals were included. The main parameter was revision rate, calculated as “revisions per 100 observed component years” to allow adjusted direct comparison of different datasets.

**Results:**

Of 16 clinical studies that met the inclusion criteria, 9 originated from the implant developer's hospital. In the clinical studies category, publications from the developer's hospital suggested considerably lower revision rates than the other datasets. In fact, the values quoted were 5.5 times below the average of all other studies, and 9.51 times lower than in the Australian arthroplasty register. These differences are statistically significant.

**Interpretation:**

The cementless Taperloc stem is an implant that shows good performance regarding revision rates in registry data and in clinical studies. However, the excellent results published by the developer's clinic are generally not reproducible by other surgeons. In terms of reference data, registry data are able to make an important contribution to the assessment of clinical sample-based studies, particularly regarding evaluation of the extent to which published results are reproducible in daily routine.

The Taperloc hip stem is a commonly used arthroplasty product that has been used worldwide for many years. This implant was developed in cooperation with Dr Richard H. Rothman of the Rothman Institute, PA, USA and is marketed by Biomet Orthopedics Inc. It is available as a cemented and an uncemented variant.

The scope of the EU Commission's EUPHORIC (European Public Health Outcome Research and Indicator Collection) project, included an examination of the validity of sources of information available for outcome quality assessment of medical devices. This involved comparative and comprehensive evaluation of various literature sources such as peer-reviewed journal publications, meta-analyses, and registry data. The present study uses survival rate as the primary parameter for the assessment of long-term results.

A major consideration in the evaluation of clinical literature by the readers of such publications is their expectation to learn from the results published, and to be able to reproduce these results. Factors such as patient selection, the fact that most studies are conducted in specialized centers of excellence, or possible publication bias may have a considerable influence on the outcome.

Since the aim of national arthroplasty registries is to cover all cases occurring in a particular country as completely as possible, bias factors due to patient selection or the influence of any particular hospital can largely be excluded. These datasets are therefore well-suited to be used as reference data for the evaluation of bias factors in sample-based studies. However, in the assessment of registry data from other countries it should be taken into account that these data reflect national circumstances such as the surgical techniques used in a particular country, or the country's general healthcare system. These circumstances—in addition to the evaluation procedures—may lead to misinterpretations (Labek et al. 2008).

## Material and methods

A web-based literature search for the terms “Taperloc”, “hip arthroplasty”, “cementless”, and “stem” was performed using common resources such as PubMed and the Directory of Open Access Journals. This was followed by a manual literature search, as well as a direct request for literature from the manufacturer of the implant. A total of 21 publications were recorded and analyzed in full text (Sharkey et al. 1990, Hozack et al. 1994, Hearn et al. 1995, McLaughlin and Lee 1995, 1997, 2000, 2006, Rothman et al. 1996, Rao et al. 1998, Lewis 2000, Keisu et al. 2001a, b, Purtill et al. 2001, Sakalkale et al. 2001, Mallory et al. 2002, Abboud et al. 2004, Bezwada et al. 2004, Parvizi et al. 2004a, b).

We used the following inclusion criteria for consideration in the subsequent evaluation: (1) unambiguous identification of the implant; (2) revision rate data either presented in the text or unambiguously calculable from the data contained; (3) English-language publications in Medline-listed, peer-reviewed journals; and (4) a minimum of 30 patients examined to exclude experimental studies and studies with a main goal other than outcome, which might have an influence on patient selection.

16 publications fulfilled these criteria (Hozack et al. 1994, 1996, Hearn et al. 1995, Rothman et al. 1996, McLaughlin and Lee 1995, 1997, 2000, 2006, Keisu et al. 2001a, b, Purtill et al. 2001, Mallory et al. 2002, Abboud et al. 2004, Bezwada et al. 2004, Parvizi et al. 2004a, b).

The main evaluation criterion was the indicator “revision rate”, a variation of which, “Revisions per 100 observed component years”, was used for comparative assessment. It was applied in accordance with the Australian National Arthroplasty Register's definition (Australian National Joint Replacement Registry 2003). The methodology is a standard procedure in epidemiology and was used, for example, for cohort studies in the 1950s concerning the effect of smoking on the incidence of lung cancer and cardiovascular diseases (Doll and Hill 1956).

 In principle, this method deals with calculating a correlation between the incidence of a potential risk exposure (e.g. smoking or implantation of an artificial joint implant) and a consequential event (e.g. lung cancer or revision surgery). Since individual years of follow-up are counted, studies with a large number of patients and long follow-up periods have higher impact on the aggregated dataset than small or short-term studies. This allows direct comparison of different studies and data sources including adjustment for number of cases and follow-up period.

A value of 1 revision per 100 observed component years corresponds to a revision rate of 5% at 5 years, or a 10% revision rate at 10 years, in conventional follow-up studies. To be rated as an outlier dataset, the average value had to show a statistically significant difference in the outcome and at least a difference of 300% to the benchmark in registry datasets. The national hip and knee registers in Sweden and Denmark publish outcome from individual departments, which show deviations of up to a factor of 3 for the outlier departments (Annual Report of the Swedish Hip Register 2008, Annual Reports of the Swedish and Danish Knee Registers 2009, Danish Hip Register 2009). This deviations in outcome were rated as explicable differences in average patient service due to cumulative effects of impact factors such as surgeon's expertise, training activities of the departments, internal and external quality control activities, patient selection or the public health system.

The journal publications included were analyzed regarding their year of publication, source of publication, follow-up period, authors, geographic region, and number of cases. These data were contrasted with the results for the Taperloc implant available from the latest annual reports of national registries, which were accessed via the internet at http://www.efort.org/education/registers.aspx. Data from the Australian National Joint Registry were available. Unfortunately, the registry report does not differentiate between the individual components revised, but only represents the revision of the implant system. As opposed to the majority of clinical studies, isolated cup revisions can thus not be derived directly, so the data were adjusted for a worldwide average benchmark.

A worldwide comparison among national arthroplasty registers presenting an exact distribution of the components concerned showed that in about two-thirds of the cases of hip arthroplasty revision, the stem component was also affected. For further calculations, this rate was transferred to the data from the Australian registry (Table 1).

**Table 1.** Distribution of revision surgeries with regard to the components revised; deviations from 100% correspond to other reasons such as isolated head or inlay exchanges

Country/Register	Reference	Cup	Stem	All
Denmark	Annual Report 2006	31%	32%	32%
England & Wales	4th Annual Report	24%	26%	54%
Norway	Annual Report 2008	26%	22%	39%
Sweden	Annual Report 2008	31%	16%	47%

For all data sources, the data were pooled in a standardized way. For any parameter except follow-up time, exact values had to be included in the study.

If no specific follow-up times but follow-up periods were given, a linear distribution of cases was assumed. To determine statistical significance, 95% confidence intervals and Poisson regression analyses were calculated using the Stata MP software version 10.1 (StataCorp LP, College Station TX).

## Results

All 16 publications of clinical studies that were finally included in the detailed analysis originated from USA. 9 articles (Hozack et al. 1994, 1996, Hearn et al. 1995, Rothman et al. 1996, Keisu et al. 2001a, b, Purtill et al. 2001, Parvizi et al. 2004a, b) were published by the developer's institution (Department of Orthopedic Surgery, the Rothman Institute, Philadelphia, PA). 4 papers (McLaughlin and Lee 1995, 1997, 2000, 2006) originated from the Center for the Hip and Knee, Oshkosh, WI, and the remaining 3 publications came from other hospitals (Mallory et al. 2002, Abboud et al. 2004, Bezwada et al. 2004). 2 publications from McLaughlin and Lee (1995, 1997) appear to have been based on the same study, since they included the same number of patients, revisions, and follow-up period. According to the general procedures for metanalyses, both were included in the evaluation as independent publications.

All papers were published between 1994 and 2006, with the highest publication frequency in 2004 (4 papers), followed by 2001 (3 papers).

The studies included were published in 4 internationally renowned journals: Journal of Bone and Joint Surgery (JBJS; American and British editions), Journal of Arthroplasty, and Clinical Orthopaedics and Related Research. One study was published as an AAOS congress contribution.

None of the publications included registry data. Three papers were multicenter studies (Abboud et al. 2004, Keisu et al. 2001b, McLaughlin and Lee 2006) and all others were monocentric. The majority of papers were based on data from retrospective clinical studies. Parvizi et al. (2004b) conducted a prospective clinical study. Mallory et al. (2002) performed a meta-analysis.

The 16 publications included involved treatment of 1,929 cases in total. However, due to the large proportion of publications from the developer's institution, multiple mentions of patients in different papers cannot be excluded. The number of cases described in the individual studies varied between 30 (Hozack et al. 1994) and 312 patients (Hozack et al. 1999), with the average number of cases in the 16 studies amounting to 120 (median 105).

The follow-up periods ranged from 2.2 years (Rothman et al. 1996) to 14.6 years (McLaughlin and Lee 2006), the average follow-up period being 8.35 years (median 8).

The majority of papers came to a positive assessment of the cementless Taperloc hip stem prosthesis. There were no clearly negative statements about the product.

Almost all studies used scores as an additional criterion to measure implant efficiency, pain, and patient satisfaction. The two scores used were the Harris hip scoring system and the Charnley modified Merle D'Aubigné and Postel scale for pain and function.

Registry data were available from the Australian National Joint Replacement Registry. From 1999 to 2008, 1,638 Taperloc stems were reported to be used in combination with the Mallory head, Recap and M2a cup; these three groups did not show any statistically significant differences in outcome. The cumulative number of cases in the Australian registry accounts for approximately the same number of cases described in studies from elsewhere. However, the average follow-up period (3.2 years) was considerably lower than in clinical studies.

An analysis of the publications according to the basic data revealed a striking difference in the distribution of revision rates published. The majority of publications from the Rothman Institute stated that there were no revisions or very low revision rates, and they showed a notable divergence from registry data and other publications (Figure) (Table 2).

**Figure:**
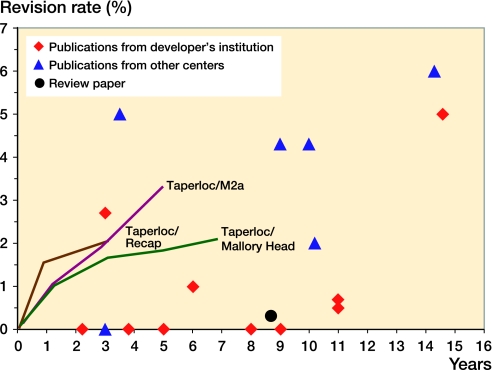
Comparison of revision rates as presented in various data sources. Lines represents adjusted data from the Australian National Arthroplasty Register 2009.

**Table 2.** Summary and description of individual publications of clinical studies included in the meta-analysis

A	B	C	D	E	F	G	H
Hozack et al.	1994	3.8	0	94	0	357	0.00
Hearn et al.	1995	3.0	2.7	30	1	90	1.11
McLaughlin et al.	1995	9.7	4.3	114	5	1,106	0.45
Hozack et al.	1996	6.1	1	105	1	638	0.16
Rothman et al.	1996	2.2	0	104	0	229	0.00
McLaughlin et al.	1997	10.0	4.3	114	5	1,140	0.44
McLaughlin et al.	2000	10.2	2	100	2	1,020	0.20
Keisu et al.	2001	5.0	0	92	0	460	0.00
Keisu et al.	2001	8.0	0	39	0	312	0.00
Purtill et al.	2001	11.0	0.5	180	1	1,980	0.05
Mallory et al.	2002	8.7	0.3	312	1	2,727	0.04
Abboud et al.	2004	3.2	0	53	0	167	0.00
Bezwada et al.	2004	3.5	4.8	168	8	588	1.36
Parvizi et al.	2004	9.7	0	86	0	830	0.00
Parvizi et al.	2004	11.0	0.7	129	1	1,419	0.07
McLaughlin et al.	2006	14.6	5.2	209	11	3,051	0.36

A Authors B Year of publication C Follow- up (years) D Revision rate (%) E No. of primary cases F No. of revision cases G Observed component years H Revisions per 100 observed component years

To determine the significance of these differences, we calculated the revisions per 100 observed component years. In a conventional meta-analysis of all clinical studies, regular impact factors showed a value of 0.22 (CI: 0.16–0.31), adjusted for the number of cases and follow-up period. Compared to the Australian registry, which showed a value of 0.57 (CI: 0.40–0.81), the probability of revisions was 2.6 times higher. This difference was found to be statistically significant.

A comparative stratification of the dataset of clinical studies in publications from the developer's institution and all other publications showed that users outside the developer's clinic had a revision rate that was 5.5 times higher. Compared to the registry dataset, the difference amounts to 9.51 fold. These differences were found to be statistically significant.

In contrast, the difference between developer-independent publications and registry datasets corresponded to a ratio of 1.73. Although this difference was found to be statistically significant, it is not clinically relevant and is within the boundaries explicable by regular impact factors (Table 3).

**Table 3.** Comparison of revision rates between clinical studies and registry data

	A	B	C	D	E	F
Developer's studies	859	4	0.06	9.51	3.17–35.2	< 0.001
Other clinical studies	1,070	32	0.33	1.73	1.06–2.86	0.03
Registry data (adjusted)	1,638	30	0.57			

A No. of primary cases B No. of revision cases C Revisions per 100 observed component years D Ratio difference to registry E CI ratio F p-value ratio difference to registry

## Discussion

There are some limitations to the validity of the data that this study is based on, as a result of estimations which were necessary to allow for the comparison of different datasets. Also, patient characteristics such as age and gender might differ in the individual datasets. Consideration of these aspects in the structured analysis was not possible because of the lack of published information and due to the inhomogeneity of studies.

For the Australian National Joint Replacement Registry, dataset allocation of the reason for revision to a component of the implant was not possible and had to be estimated. It is unlikely, however, that this would have a substantial effect on the final conclusions when differences of 951% were observed. Comparative analysis of the datasets has yielded several observations that should lead to critical scrutiny.

Systematic analysis of the data published about the cementless Taperloc stem revealed a strikingly high influence of the developer's clinic: 44.53% of all cases and 39.2% of observed component years were published by this institution. However, with regard to revision rate this dataset differs considerably in outcome from the reference data in the Australian registry and developer-independent publications. Even in a conventional meta-analysis of the data, the high influence of the developer's institution has a statistically significant effect on the overall result, which has also been demonstrated in the meta-analysis by Mallory et al. (2002).

Several points should be considered in the interpretation of the data:

*1. Influence of the developing hospital*

Developers of implants have several particularities that might influence the outcome:

- As a rule, the hospital concerned can rely on a high degree of expertise and a fundamental understanding of the product and its handling
- High personal motivation can be assumed when it comes to the thorough investigation of potential outcome-relevant flaws in the entire course of therapy and drawing the consequences.
- The final result of a THA implantation depends on a variety of factors, such as the product, instrumentation, operating technique, patient selection, etc. Since every product is developed against a specific background and based on a specific set of experiences, the product might make particular allowance for the factors prevailing at that particular hospital
- Last but not least, there are the implant designer's and the manufacturer's own interests in the success of the product.

*2. Differences in outcome and reproducibility of the results of others, i.e. the average surgeon*

Drawing on the differences in revision rate among individual hospitals in Sweden for interpretation of the divergences shows that the best departments deviate from the national average by a factor of 2 to 3 (Annual Report of the Swedish Hip Register 2008, Annual Reports of the Swedish and Danish Knee Register 2009, Danish Hip Register 2009). Since one of the most important reasons for the success of the Scandinavian registries is the reduction of individual hospitals' variances in revision rates through the higher-than-average improvement of low-performing hospitals, one can assume that there is a higher variation between results in individual hospitals in the US. The extent to which the other hospitals that have published follow-up studies on the Taperloc stem differ from the developer's institute as regards expertise should be critically examined. In any case, the data available allow us to make the conclusion that the outcome data achieved by the developing hospital are not reproducible by the average user worldwide.

Thus, the high influence of the developing institution could have a relevant impact on the average results published about the product.

As a rule, the circumstances surrounding journal publication are favorable for publishing studies from the developing institution:

- It can be assumed that implant developers have a strong commitment and a high scientific interest and motivation regarding follow-up so that there is sufficient material for journal publications
- Developing hospitals are the first to have access to new implants and are thus the first to be able to publish results. These publications are of innovative quality and are therefore attractive for journals
- As a rule, development teams closely cooperate with implant manufacturers, thus having access to resources that, among other things, can be used in follow-up and publications
- Since studies are also used in the marketing of the respective implant, manufacturers and developers are much more interested in publication than is usually the case for projects based exclusively on academic motivation, or for average users.

The majority of journal publications report a lower probability of revision than registry data. In this respect, a fundamental difference in the basic data must be taken into account: clinical studies are based on samples, which implies that any transference of results involves potential confounders. For example, the vast majority of publications originate from specialized centers, which are not representative of the worldwide or national average in all aspects. As opposed to this, registry data contain almost all surgeries performed in a country and therefore comprise the entire range of treatment, thus reducing bias factors and allowing better generalization in the area covered by the registry. The transference of results to other countries naturally creates confounders due to the local background, but its influence is considerably lower than in meta-analyses of clinical studies.

For the cementless Taperloc stem, there are no sufficient data currently available from national registries other than the Australian one. For other implants, however, such comparisons have shown that deviation from the average outcome value of the respective implant by a factor of 3 is not reached in any case.

Regarding the deviations in revision rates between individual hospitals within a certain country, without specifying factors such as implant selection, surgical techniques, the profile of the department, or other confounders, and between countries regarding the outcome achieved with the same implant, deviations by a factor of 3 appear to be explicable.

Thus, publications by authors who are not directly involved in the process of development of an implant on average show results that are reproducible in the registry, even though the average revision rates are below those of the Australian registry. The average revision rates published by the developer's institution, however, do not appear to be reproducible by other users. On average, they are approximately 9.5 times higher in the Australian register, and about 5 times higher with other users in the USA. The specific circumstances of a developing hospital therefore seem to affect the results.

*3. Basic characteristics of the datasets that are being compared*

In any evaluation, special consideration has to be given to the particular features inherent to the various datasets involved. As mentioned before, sample-based studies are usually conducted in specialized centers and subject to several influencing factors, such as the study design, patient selection, etc. However, the different factors influencing the results of sample-based studies are frequently not or only insufficiently apparent from the publications while they may have a relevant impact on the study results and lead to a remarkable variance in outcomes. This reduces the reliability of the data for conclusions, particularly when individual centers are overrepresented.

Registry data can be a valuable supplement for evaluation of the revision rates to be expected by the scientific community. Due to their higher degree of standardization and completeness in case recording at the national level, their validity is on average superior to that of sample-based clinical studies. Optimum reference values, however, can only be obtained within the coverage area of a register since the circumstances under which the data have been collected are always reflected in the results. Setting up of a registry should therefore be aspired to in every country.

It would generally be desirable if a large number of hospitals and surgeons were to undertake structured patient follow-ups and publish the results. This could at least reduce confounders due to individual circumstances in different hospitals.
